# Advancements and Challenges in High-Capacity Ni-Rich Cathode Materials for Lithium-Ion Batteries

**DOI:** 10.3390/ma17040801

**Published:** 2024-02-07

**Authors:** Mehdi Ahangari, Benedek Szalai, Josue Lujan, Meng Zhou, Hongmei Luo

**Affiliations:** Department of Chemical and Materials Engineering, New Mexico State University, Las Cruces, NM 88003, USA; mahani92@nmsu.edu (M.A.); szalaib@nmsu.edu (B.S.); jluja111@nmsu.edu (J.L.)

**Keywords:** Ni-rich cathode, surface modification, elemental doping, concentration gradient

## Abstract

Nowadays, lithium-ion batteries are undoubtedly known as the most promising rechargeable batteries. However, these batteries face some big challenges, like not having enough energy and not lasting long enough, that should be addressed. Ternary Ni-rich Li[Ni_x_Co_y_Mn_z_]O_2_ and Li[Ni_x_Co_y_Al_z_]O_2_ cathode materials stand as the ideal candidate for a cathode active material to achieve high capacity and energy density, low manufacturing cost, and high operating voltage. However, capacity gain from Ni enrichment is nullified by the concurrent fast capacity fading because of issues such as gas evolution, microcracks propagation and pulverization, phase transition, electrolyte decomposition, cation mixing, and dissolution of transition metals at high operating voltage, which hinders their commercialization. In order to tackle these problems, researchers conducted many strategies, including elemental doping, surface coating, and particle engineering. This review paper mainly talks about origins of problems and their mechanisms leading to electrochemical performance deterioration for Ni-rich cathode materials and modification approaches to address the problems.

## 1. Introduction

In recent decades, the urgent global issues of ever-increasing fossil fuel demand and the alarming threat of global warming have spurred the development of renewable and green energy sources [1]. Within this array of sustainable energy options—for example, hydroelectric, solar, tide, or wind, which all contribute to the global energy needs—rechargeable lithium-ion batteries (LIBs) have prominently emerged as the most sought-after for numerous practical applications, decisively spearheading the electrification of vehicles. This preference arises from their distinctive blend of characteristics, including their lightweight design, impressive power output, substantial energy capacity (ranging from 250 to 693 Wh L^−1^ and 100 to 265 Wh kg^−1^), relatively long service life, desirable rate capability, safety features, and minimal environmental impact. Currently, LIBs have achieved widespread success in small-scale portable consumer electronic devices such as cellular phones and laptop computers [2,3]. However, when it comes to utilization of the current state-of-the-art LIBs in more substantial applications, such as passenger electric vehicles (EVs), hybrid electric vehicles, and large-scale energy storage systems, there are formidable challenges and requirements (as detailed in Table 1) that must be meticulously addressed to compete with traditional internal combustion engine vehicles and receive wider consumer acceptance. These challenges encompass the imperative to meet demanding criteria, such as fast charging and rating capabilities, elevated energy, power density for starting, accelerating, and uphill driving, cost-effectiveness, battery safety under harsh conditions at elevated temperatures, and prolonged lifespan [4]. One way to reduce costs is to utilize high gravimetric and volumetric energy density materials, thereby requiring a smaller number of needed materials. In addition to driving range, charging time is also central to customer experience [5,6,7,8,9,10].

**Figure 1:**
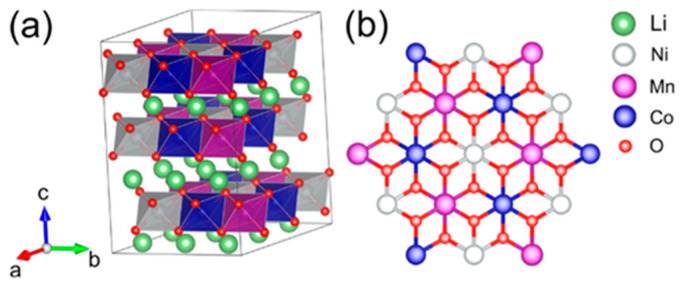
(**a**) Schematic view of the bulk structure of NCM111 with R-3m space group, and (**b**) illustration depicting the ion-ordering in NCM111 TM layers. Reprinted from Ref. [18] with permission from the American Chemical Society.

The electrochemical characteristics of LIBs are primarily governed by the properties of their cathode materials. While every component of the battery system plays a vital role in achieving optimal performance and continues to be the focus of extensive research, a notable technical bottleneck in LIBs technology pertains to the limited capacity of cathode materials, typically falling below 270 mAh g^−1^. This inferior capacity stands in contrast to the more robust capacity of graphite, SnO_2_, Sn, and Si anode materials, which can reach up to 350, 782, 994, and 4200 mAh g^−1^, respectively [12]. In order to resolve the capacity shortfall exhibited by cathode materials, immense efforts have been directed on research and development endeavors aimed at creating new materials and refining existing materials that combine high-capacity and high-voltage. Cathode materials are typically categorized into the three most prominent classes: (1) Layered oxide LiMO_2_ (M = Mn, Co, and Ni) structure, (2) Spinel-framework LiM_2_O_4_ (M = Mn, Co, and Ni) structure, and (3) Olivine LiMPO_4_ (M = Fe, Mn, Co, and Ni) structure [13]. These categories exhibit varying actual capacities, typically falling within the range of 120 mAh g^−1^ to 210 mAh g^−1^ [14,15,16]. Among the various materials presently available explored as potential candidates for battery cathodes, layered transition metal oxides (LTMOs) have emerged as particularly promising options. Layered oxide materials (for example LiCoO_2_ and LiNi_x_Co_y_Mn_z_O_2_) with a layered lattice α-NaFeO_2_ hexagonal structure and R-3m space group play a crucial role in facilitating the reversible (de)intercalation of lithium ions. In this structure, lithium ions and TM cations are placed within octahedral 3a and 3b sites, respectively, while oxygen anions occupy the octahedral 6c sites, showing a cubic close-packed arrangement (as depicted in Figure 1) [17].

The evolution of Ni-rich cathode materials traces its roots to the constrained development of LiCoO_2_ electrodes. Despite the excellent performance of LiCoO_2_, limitations such as practical delivery of only 50% of the theoretical capacity (140 mAh g^−1^), high cost, and the material’s toxic property, hinder its application in EVs. As a response, rigorous research endeavors have been undertaken to replace Co with alternative elements such as Ni and Mn in cathode hosts. However, the absence of Co in the layered oxide structure often results in structural instability at low-temperature operations and lower electronic conductivity [19,20]. N. Zhang et al. [21] reported that Co helps Ni and Li ordering, and in Co absence, more Ni and Li ion-exchange sites are possible. Also, Mn provides structural stability, as its oxidation state is +4 throughout electrochemical cycling [22]. Consequently, by taking partial advantage of the high capacity of LiNiO_2_, the layered character of LiCoO_2_, and the low cost and thermal stability of LiMnO_2_, LiNi_x_Co_y_Mn_z_O_2_ (NCM) cathode materials have appeared as viable contenders to substitute the traditional LiCoO_2_ cathode, addressing some of the inherent challenges associated with Co-based formulations [23,24,25]. Ni-rich layered oxide cathode materials (x > 0.5) have attracted great interest as the leading candidates in this domain. This enthusiasm is primarily fueled by their exceptional attributes, including a superior actual capacity exceeding 170 mAh g^−1^, a volume change by less than 2% during Li insertion/extraction, and a higher average operating cutoff potential surpassing 3.4 V [26,27,28,29]. While Ni-rich cathode materials have received great attention for their remarkable attributes, such as high capacity and energy density (exceeding 600 Wh kg^−1^), they are susceptible to a range of significant drawbacks. These issues include premature capacity degradation, severe structural and thermal instability, limited calendar life, electrolyte decomposition, intrinsic low-rate capability, rapid voltage decay leading to sluggish Li^+^ ion transport dynamics, and safety concerns. These intrinsic defects have posed significant obstacles to their widespread commercialization, particularly in the context of EVs [30,31,32,33,34]. In this review, we delve into the main mechanisms contributing to the failure of Ni-rich cathode materials in LIBs. By exploring these mechanisms in depth, we shed light on key strategies aimed at enhancing both the electrochemical and thermal properties of these materials.

## 2. Origin of Problems

**Figure 2:**
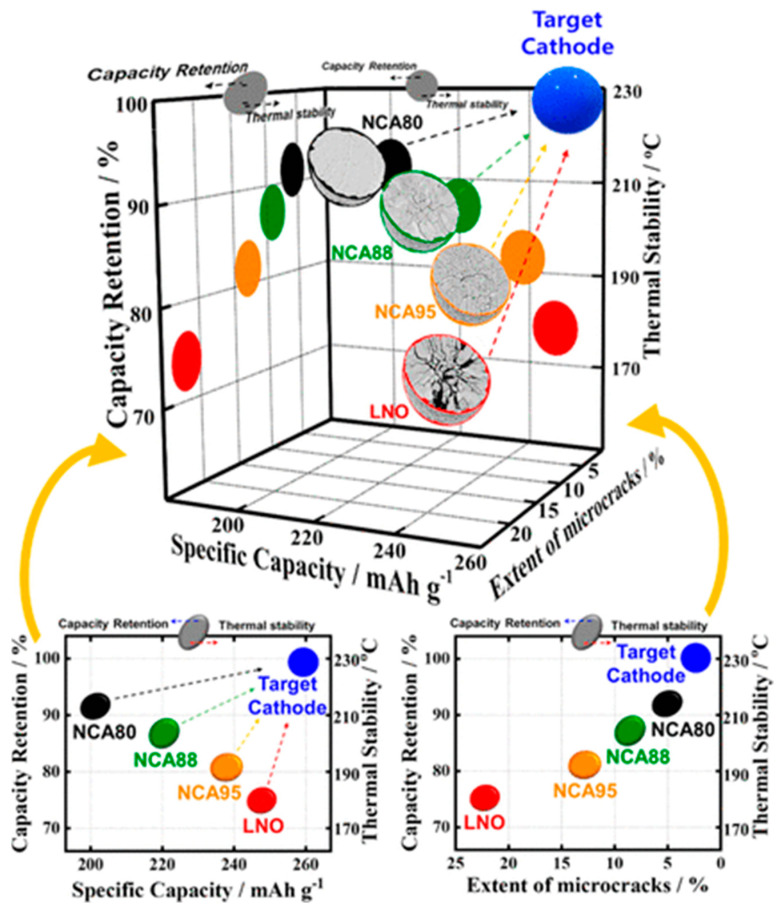
Illustration depicting the relationship among specific capacity, capacity retention, microcrack propagation, and thermal stability in Ni-enriched NCA and LNO cathodes. Reprinted from Ref. [36] with permission from the American Chemical Society.

The broad applications of Ni-rich cathode materials in the industry have faced significant challenges, primarily stemming from two issues: performance degradation and safety concerns. C. Yoon et al. [35] conducted a study highlighting that an escalation in Ni concentration within NCM cathode materials resulted in a notable decline in capacity retention. According to their research, the increase in Ni content in NCM cathode materials correlated with an elevated discharge capacity, ranging from 160 mAh g^−1^ in NCM111 to surpassing 240 mAh g^−1^ in LiNiO_2_. However, it is essential to highlight that capacity retention experienced a decrease, diminishing from 97% for NCM111 to 70% for LiNiO_2_. Another investigation by G. Nam et al. [36] indicated an augmentation in Ni content in layered oxides, accompanied by sacrifices in electrochemical performance and thermal stabilities due to the weaker Ni−O bonds destabilizing the crystal structure and increasing the anisotropic volume change upon Li-extraction/insertion, leading to a significant increase in microcrack propagation (refer to Figure 2). In Ni-rich active materials, key challenges fall into two categories: crystal structure-driven degradation, and instability of the electrode material interface near the surface. One major issue contributing to structural degradation is Li/Ni cation mixing which appears particularly in NCM materials with a Ni content more than 80%. This mixing leads to various deteriorations in the cathode material, such as pulverization, phase transition, and microcrack propagation, which makes it difficult to reach high capacity retention in cycling and rating performance. Regarding electrode-electrolyte reaction, electrolyte decomposition and gas evolution are significant consequences, posing thermal instability and safety hazards, especially in EVs. These problems will be thoroughly discussed in this section, providing valuable insight for researchers studying cathode materials for LIBs.

### 2.1. Understanding the Structural Instability

**Figure 3:**
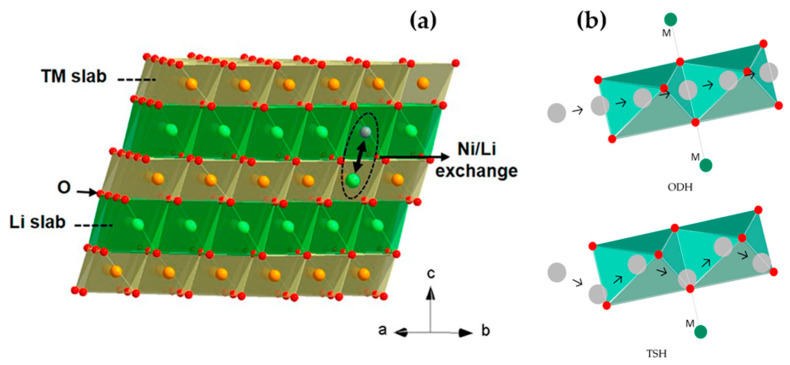
(**a**) The α-NaFeO_2_ type structure of layered NCM materials, featuring one pair of Li/Ni exchange. Reprinted from Ref. [38] with permission from American Chemical Society, and (**b**) A schematic representation is provided, elucidating the Li ion diffusion pathways through ODH and TSH. Green, red, and grey dots stand for TM ions, oxygen, and Li ions, respectively [40].

The cathodes of LIBs function through the reversible insertion and extraction of Li ions. This process is expected to transpire without inducing any significant permanent alterations to the crystal structure, as these cathodes need to withstand hundreds to thousands of charging and discharging cycles. In Ni-enriched cathode materials, the coexistence of mixed-valent Ni^2+^ and Ni^3+^ leads to partial Li/Ni structural disordering. Li/Ni cation mixing degrades the capacity of Ni-rich cathode materials and hinders the mobility of both Li and Ni ions (Figure 3a). The exchange between Ni and Li occurs during the synthesis process and cycling in LTMO structures. In these structures, Ni^2+^ preferentially occupies the interlayer Li 3b site over Ni^3+^, as Ni^2+^ and Li^+^ have similar sizes (0.69 Å and 0.76 Å, respectively) and a lower energy barrier for Ni^2+^ ions’ migration from their native 3b to Li^+^ 3a sites. From an ionic model perspective, the layer structure of LiNiO_2_ is expressed as [Ni^3+^]_3a_−[O^2−^]_6c_−[Li^+^]_3b_−[O^2−^]_6c_. Ni substitution in the Li 3b layer accompanies a change in Ni valence to maintain the electroneutrality principle, and is represented as [Ni^2+^]_3a_−[O^2−^]_6c_−[Ni^2+^]_3b_−[O^2−^]_6c_ [37]. This promotes Li^+^/Ni^2+^ exchange, with a higher probability compared to the lower likelihood of such exchange for Co^3+^ and Mn^4+^.

The explanation for the structural instability induced by the exchange of Li and Ni can be elucidated as follows. As shown in Figure 3b, Li ions diffuse within the layered oxide structure through two pathways: The first involves Li ions moving from one octahedral site to the next octahedral site by hopping directly through the oxygen dumbbell directly, termed oxygen dumbbell hopping (ODH), with an activation barrier ranging from 0.75 to 0.91 eV. The second pathway entails Li ions diffusing from one octahedral site to the next octahedral site by hopping through a divacancy left by Li diffusion to an intermediate tetrahedral site surrounded by TMs, known as tetrahedral site hopping (TSH). The corresponding energy barrier for TSH ranges from 0.36 to 0.54 eV. These values are influenced not only by the electrostatic interaction between Li ions in the activated state and the TM cations but also by the strain effect, which encompasses the size of the saddle point for the oxygen dumbbell in ODH and the size of the tetrahedral site for TSH [38,39].

The presence of anti-site Ni^2+^ hampers Li ion diffusion in the Li-layer through three mechanisms: Firstly, as Li ion migration in LTMO_2_ occurs via 2D diffusion within the Li layer, the presence of anti-site Ni^2+^ disrupts the Li ion diffusion path. Secondly, the higher charge of anti-site Ni^2+^ should result in stronger electrostatic repulsion of migrating Li ions, consequently leading to lower Li ion mobility. Thirdly, the activation energy of both ODH and TSH diffusion is closely associated with the strain effect, determined by the c-lattice parameter (Li slab space). Due to the stronger interaction of O−Ni^2+^−O compared to that of O−Li^+^−O, the presence of anti-site Ni^2+^ reduces the Li slab space, leading to a higher activation barrier for lithium diffusion [38]. The microscopic mechanisms can be explained in the following ways: (1) When Ni ions with valence states of 2+, 3+, and 4+ are replaced with an Li ion, the local coulombic interaction of the corresponding TM ions changes, and the network of super-exchange interactions among the TM ions is also disrupted due to the absence of localized d-orbital electrons in the anti-site Li ions. Thus, both the coulombic interaction and super-exchange interaction contribute to the distortion force, resulting in anisotropic stress within the bulk structure [38,41,42,43]. (2) During electrochemical cycling, the anti-site Ni ions would gradually migrate to the particle surface due to the low energy barrier for Ni^2+^ migration (∼0.25 eV). This migration process results in Ni depletion in the bulk, leading to structural instability, and consequently, cathode voltage and capacity fade [44].

**Figure 4:**
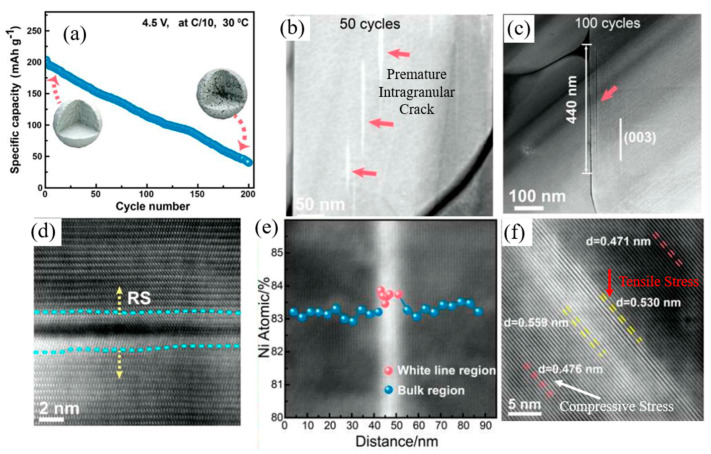
(**a**) Cycling performance of NCM811 at a rate of 0.1 C, (**b**,**c**) low-magnification STEM-HAADF images illustrating the progression of intragranular crack development in cycled NCM811, (**d**) STEM-HAADF images at atomic resolution taken on a cycled NCM811 cathode in a <010> zone projection, (**e**) quantitative atomic percentage of Ni in the white line region (marked with red balls) and the normal bulk region (indicated by blue balls), and (**f**) High-resolution STEM-HAADF image displaying lattice distortion in the white line region. Reprinted from Ref. [45] with permission from Elsevier.

Lin et al. [45] provided evidence of the occurrence of lattice distortion in NCM811 during cycling, a phenomenon that led to the propagation of microcracks and subsequent capacity decay. The researchers underscored that the generation of intragranular cracks originating from the bulk of primary particles was accountable for the degradation in electrochemical performance observed in NCM811 (Figure 4a). In their findings, they detailed the development of a premature intragranular crack within the NCM811 primary particle. As shown in Figure 4b, this crack exhibited a length of 50 nm along the (003) plane, identified as the preferential direction for Li/Ni anti-site formation. Notably, the primary particle underwent a phase transformation from a layered structure to a rock-salt structure, extending to 440 nm (Figure 4c,d), indicative of the growth of the transformed structure region. The structural evolution was elucidated by the reduction of Ni ions to a lower state and the involvement of oxygen in redox reactions. This resulted in the formation of additional oxygen vacancies and a lowered migration barrier for TM cations in the Li layer, leading to the aggravation of rock-salt domain [46,47]. Consequently, Li^+^ sites were occupied by Ni^2+^. With an increase in the cycling number, an augmented occurrence of Li/Ni anti-site defects at the crack edge and the aggregation of Ni ions in the Li ions diffusion channel was observed, confirmed by EDS mapping shown in Figure 4e. This led to an elevation in the columbic repulsion of cations due to the higher charge state of Ni^2+^ compared to Li^+^, coupled with increased lattice distortion by tensile strain due to the expansion of d-spacing of the (003) plane. As the cycling continued, the tensile stress in the deformation region surpassed the tensile limit of the transformed structure, resulting in the fracture of the distorted lattice (Figure 4f). Notably, the fragility of the rock-salt phase compared to the layered phase played a crucial role in the fracture mechanism, leading to lattice rupture along the (003) plane.

Li/Ni lattice disordering induces partial interference disruption in the (003) plane, resulting in a reduction of the intensity of the (003) peak. Concurrently, the constructive interference of peaks in the (104) plane increases, leading to a decrease in the I(003)/I(104) ratio. Based on XRD analysis, the increase in Ni content did not change the crystal structure of materials (hexagonal α-NaFeO_2_), except for the I(003)/I(104) ratio, which decreased in higher Ni concentration materials. It is worth mentioning that the I(003)/I(104) ratio is indirectly a sign of Li/Ni cation mixing in the lithium layer and a ratio of more than 1.2 signifies a well-ordered structure. Table 2 provides I(003)/I(104) ratio measurements for a range of NCM cathode materials.

**Figure 5:**
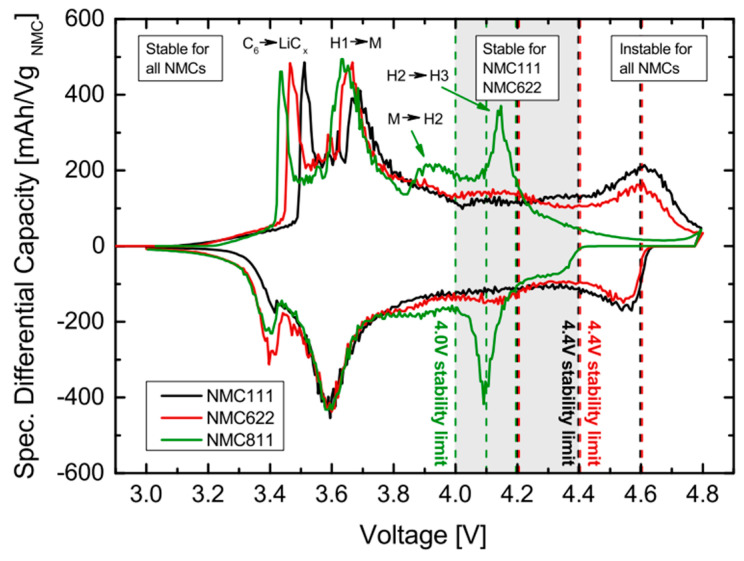
Differential capacity against cell voltage at 0.1 C-Rate (3rd Cycle), highlighting peaks aligned with distinct phase transitions (H1, H2, H3, and M). Reprinted from Ref. [54] with permission from ECS.

Destabilization of mechanical integrity in cathodes is attributed to the large and nonuniform lattice contraction, intensified by the abrupt H2 → H3 phase transition. This phenomenon leads to the formation of numerous microcracks, particularly noticeable with an increased Ni fraction. In Ni-enriched cathode materials, structural degradation is characterized by a transition from the layered phase (R-3m) to a spinel-like phase (Fd-3m) and a rock-salt phase (Fm-3m) [49,50]. In these cathode materials with x ≥ 0.8, a sequence of phase transitions, including the transition from the layered structure (H1) to a monoclinic phase (M) and two hexagonal phases (H2 and H3), takes place. The phase transition from H2 to H3, occurring above 4.15 V, has been identified as a significant contributor to rapid capacity fading in NCM and NCA materials, resulting in an abrupt shrinkage of approximately 0.3 Å in the c-direction. During discharge, the transition from H3 to H2 causes an equal expansion in the lattice. As this abrupt anisotropic volume change repeats during cycling, the structural stability of the cathode is gradually compromised, ultimately leading to capacity fading [51,52]. Differential capacity vs. voltage profiles (dQ dV^−1^) for NCM materials with different Ni molar ratios confirm that phase transitions occur at higher Ni fractions, where the NCM811 curve deviates significantly from the corresponding ones for NCM111 and NCM622 (Figure 5). Up to 3.8 V, there is a peak at around 3.7 V during charging for all samples. For higher potential, the curve for NCM811 exhibits sharp peaks due to phase transition from M → H2 at around 3.85 V, and H2 → H3 at around 4.15 V. As cycling progresses, NCM111 demonstrates the most stable structure compared to Ni-rich samples. The absence of phase transition H2 → H3 in lower Ni concentration materials reflects the good reversibility and stability of the host structure [48]. During this phase transition, TMs migrate to vacant Li sites, accompanied by changes in lattice constants and volume with each charge–discharge cycle, leading to the accumulation of local stress concentrations within particle boundaries, ultimately resulting in crack formation. The cracks propagate through the cathode particle, allowing electrolyte solution infiltration. Upon contact with the exposed surfaces of the cracked cathode, the electrolyte solution triggers further deterioration of the structural and electrochemical properties. This deterioration is exacerbated by the reaction with highly unstable reactive Ni^4+^ (resulting from the reduction of Ni^4+^ to Ni^2+^, accompanies by O_2_ release) and the continuous accumulation of a NiO-like impurity layer [53].

**Figure 6:**
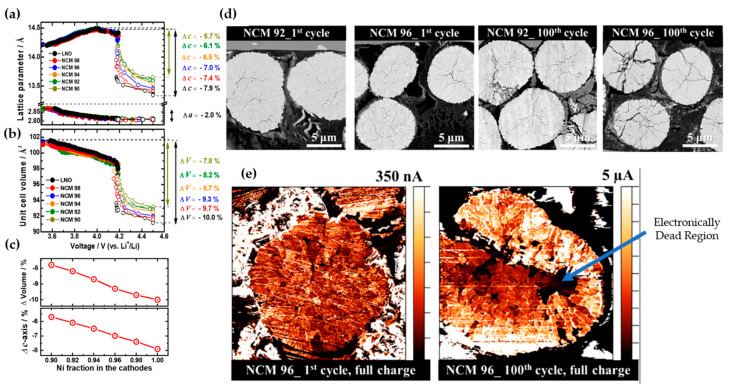
Comparative analysis of (**a**) a and c-axis, (**b**) unit cell volume variations in Li[Ni_x_Co_(1−x)/2_Mn_(1−x)/2_]O_2_ cathodes with varying Ni fractions (x = 0.9, 0.92, 0.94, 0.96, 0.98, and 1.0), (**c**) investigation into the maximum contraction along the c-axis and unit cell volume during charging, correlating with the Ni fraction in the cathodes, (**d**) cross-sectional SEM images depicting the structural evolution of Li[Ni_x_Co_(1−x)/2_Mn_(1−x)/2_]O_2_ cathode materials at (x = 0.92 and 0.96) during the 1st and 100th charged states, and (**e**) SSRM images illustrating the evolution of an NCM96 particle following the 1st and 100th complete charging cycles. Reprinted from Ref. [53] with permission from the American Chemical Society.

Kim et al. [53] investigated the influence of Ni enrichment on the volume, lattice parameters, and microstructure of Ni_x_Co_(1−x)/2_Mn_(1−x)/2_ (x = 1, 0.98, 0.96, 0.94, 0.92, and 0.90). As shown in Figure 6a, the a-axis lattice parameter consistently decreased with the NCM composition, up to 2%. In contrast, the c-axis parameter initially expanded and then abruptly contracted beyond 4.2 V due to the H2 → H3 phase transition. The contraction of the a-lattice parameter was limited to about 2%, while the reduction for the c-axis parameter was more significant, resulting in highly anisotropic volume contraction. Figure 6a illustrates a pronounced contraction in the c-axis parameter for LiNiO_2_ compared to NCM90 (−7.9% and −5.7%, respectively). The unit cell volume displayed a similar trend, diminishing beyond 4.2 V, with increased volume changes as the Ni fraction rose, measuring −7.8% for NCM90 and −10% for LNO (Figure 6b). In Figure 6c, the ∆c and ∆V curves indicated that the primary cause of volume contraction in cathodes with higher Ni fractions was the reduction along the c-direction during the H2 → H3 phase transition. This reduction induced local stress concentrations at the boundaries of primary particles, serving as sites for crack nucleation. Cross-sectional SEM images of two samples, NCM92 and NCM96, distinctly revealed mechanical damage induced by the H2 → H3 phase transition during cell cycling. Microcracks propagated to the surface of NCM96, whereas in the case of NCM92 cathodes, microcrack propagation was arrested before reaching the particle surface due to lower volume changes (Figure 6d). The scanning spreading resistance microscopy (SSRM) technique was employed to determine that the electronically dead region (near-zero electronic conductivity) of NCM96 was fully charged to 4.3 V after the first and 100 cycles. Dark regions were observed, indicating electronically inactive areas due to electrolyte penetration and surface degradation, accentuated by microcrack propagation (Figure 6e).

The mechanism of the phase transition in Ni-enriched materials can be explained by the level of removal of Li from insertion oxides or SOC through various mechanisms. Initially, lithium removal occurs randomly, but as vacancies accumulate, the remaining Li ions may undergo an ordering transition. In most oxides, Li ionizes to Li^+^ during removal, creating electronic holes in the host material. This introduction of electronic holes can trigger phase transitions through various pathways. If, at a specific Li composition, the material exhibits insulating or semi-conducting behavior, Li removal can induce a transition to a metallic state once a sufficient number of mobile holes (electrons) are generated in the host. The shift in valence of ions within the host material may alter the short-range interaction between them, either through size changes or shifts from ionic to more covalent bonding. This evolution in short-range interaction, coupled with the change in electrostatic energy resulting from shifts in valence and the removal of ions, can lead to structural modifications in the host [55]. For example, in the case of NCM811, substantial delithiation (charging) resulted in the lattice parameters a and b of Li_x_NCM811 shrinking for 1.00 > x > 0.25, followed by a slight expansion for 0.25 > x. Meanwhile, the c-lattice parameter initially increased for 1.00 > x > 0.50 and then decreased for 0.25 > x [56]. The microcrack propagation due to volume changes in cathode materials, which is more pronounced particularly in terms of density and width in Ni-rich cathode materials, could serve as channels for electrolyte penetration, exposing the particle interior to deleterious electrolyte reactions.

**Figure 7:**
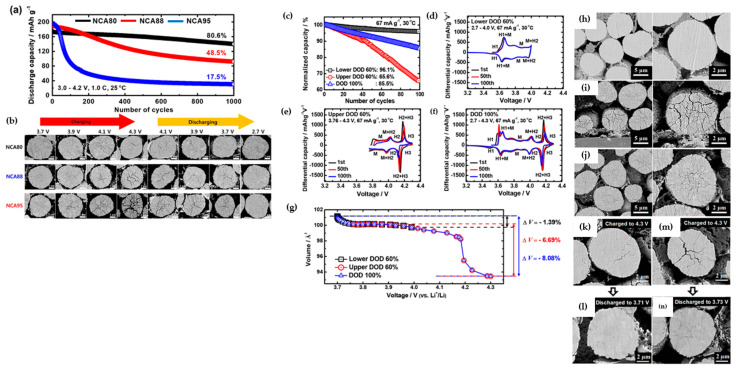
(**a**) Comparative long-term cycling performance evaluation of NCA80, NCA88, and NCA95 cathodes in pouch-type full-cells with graphite as the anode, (**b**) comparison of cross-sectional SEM images showcasing the NCA80, NCA88, and NCA95 cathodes at different SOC levels. These include charged states at 3.7, 3.9, 4.1, and 4.3 V, as well as subsequent discharged states at 4.1, 3.9, 3.7, and 2.7 V during the initial cycle. Reprinted from Ref. [36] with permission from the American Chemical Society, (**c**) performance of NCA95 cathodes during cycles, subjected to charging and discharging across varied DOD ranges, graphs illustrating dQ dV^−^^1^ curves for NCA95 cathodes undergoing cycling at (**d**) 60% lower DOD, (**e**) 60% upper DOD, and (**f**) 100% DOD over multiple cycles, (**g**) variations in unit cell volume during the charging process of NCA95 cathodes at various DOD ranges, SEM images depicting cross-sectional views of NCA95 cathodes following 100 cycles: (**h**) 60% lower DOD, (**i**) 60% upper DOD, and (**j**) 100% DOD, SEM cross-sectional images capturing the initial cycle of NCA80 and NCA88 cathodes: (**k**) NCA80 charged to 4.3 V, (**l**) NCA80 charged to 4.3 V and discharged to 3.71 V, (**m**) NCA88 charged to 4.3 V, and (**n**) NCA88 charged to 4.3 V and discharged to 3.73 V. Reprinted from Ref. [58] with permission from the American Chemical Society.

**Figure 8:**
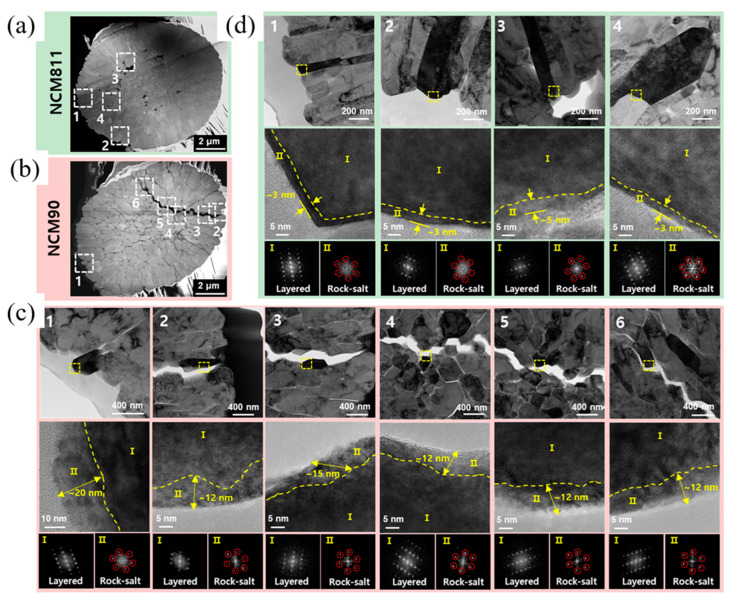
Dark-field STEM images depicting fully discharged states of (**a**) NCM811 and (**b**) NCM90 cathodes after 1000 cycles. The yellow dashed squares in the images represent magnified TEM images and high-resolution TEM images. FFT patterns from regions I and II are provided for the (**c**) NCM811, and (**d**) NCM90 cathodes. Reprinted from Ref. [59] with permission from the American Chemical Society.

G. W. Nam et al. [36] investigated the effect of level of SOC in structural degradation for Li[Ni_1−x−y_Co_x_Al_y_]O_2_ cathodes with compositions (1 − x − y = 0.8, 0.88, and 0.95). Based on their research, NCA80 retained a substantial fraction of the H2 phase even though it was charged to 4.3 V, while the NCA88 and NCA95 H2 → H3 phase transition was completed at lower potential and at higher acceleration rate, which did not allow the mechanical strain to dissipate and led to localized strain concentration, from which microcracks nucleated along the grain boundaries. The areal fraction was 5% for NCA80, 14.0% for NCA88, and 25.7% for NCA95 after charging to 4.3 V, and the length and density of cracks were visibly increased. It was concluded from these measurements that NCA95 fully charged to 4.3 V experienced pulverization into clumps of constituent primary particle due to a high anisotropic volume change, and it was one of the main culprits for its cycling deterioration. Figure 7a,b explain the reason behind different capacity deterioration behaviors in NCA compositions. When NCA electrodes were charged and discharged, the microcracks were not completely closed, showing irreversible microstructure evolution, especially for NCA95. The severity of microcracking in the NCA88 and NCA95 was significant enough to permit electrolyte penetration, rendering the particle susceptible to electrolyte attack and resulting in an accelerated rate of capacity fading due to the reaction with Ni^4+^ species and the formation of NiO-like rock-salt phase on the cathode surface [57]. Park et al. [58] conducted a comprehensive investigation aimed at elucidating the factors contributing to capacity fading in NCA95, NCA88, and NCA80 materials. These materials underwent cycling under various conditions, including the upper range (3.76–4.3 V) and lower depths of discharge (DOD) at 60% (2.7–4.0 V) and 100% DOD (2.7–4.3 V). The results, illustrated in Figure 7c, revealed a more pronounced capacity retention decline at the upper DOD (65.6%) compared to other conditions. Analysis of the dQ dV^−1^ curves for NCA95 (Figure 7d–f) highlighted the significant influence of DOD levels on capacity retention. Specifically, the redox peaks corresponding to the H2 → H3 phase transition exhibited a faster decay in height during cycling at the upper 60% DOD than at 100% DOD. Conversely, the redox peaks associated with the H1 → M phase transition at the lower 60% DOD remained relatively stable, indicating the host structure’s robustness in this DOD range. Figure 7g depicted the volume change of NCA95 during cycling, revealing the highest value for cycling at 100% DOD, conventionally associated with microcrack propagation in particles. Conversely, the areal fraction of microcracks at the upper DOD was twice that at 100% DOD, as can be seen in Figure 7h–j. These findings emphasized the critical influence of DOD width and limit on microcrack resistance. Extending the investigation to NCA88 and NCA80 samples, cross-sectional SEM images (Figure 7k–n) after 100 cycles at the upper DOD confirmed that materials with a higher 80% Ni fraction experienced notable impacts on cycling performance due to DOD limit and width. The authors concluded that the primary cause of severe microcrack propagation at the upper DOD was attributed to the insufficient relief of stress generated by the H2 → H3 phase transition at 3.76 V, necessitating additional lithiation. The thickness of the NiO-like rock-salt phase is highly dependent on the composition of cathode materials. According to transmission electron microscopy (TEM) images (Figure 8a,b) provided by Park et al. [59], surface damage for Ni_0.9_Co_0.05_Mn_0.05_ (NCM90) fully discharged after 1000 cycles appeared as residual microcracks through which the electrolyte can infiltrate, and were discernible in the cathode particle, in contrast to hairline cracks in NCM811. Simultaneously, the thickness of the damaged layer formed on the exterior surfaces and near microcracks attributed to the rock-salt impurity and reactivity of the cathode with the electrolyte was higher for the NCM90 than NCM811 particle, at 20 and 3 nm, respectively (Figure 8c,d). The increased thickness of the rock-salt impurity, stemming from microcrack propagation and electrolyte infiltration, has the potential to elevate the charge transfer resistance associated with Li ion diffusion. This phenomenon can contribute to increased polarization and kinetic losses in capacity, particularly at high C-rates.

### 2.2. Electrode-Electrolyte Interface Instability

**Figure 9:**
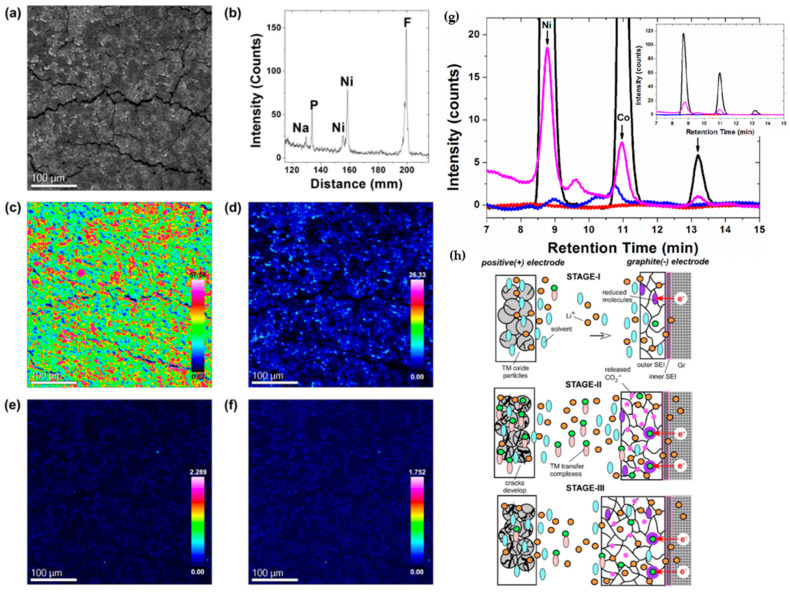
Examination of the anode surface following 500 cycles through EPMA analysis. (**a**) Backscattered electron image, (**b**) EPMA spectrum. Elemental mappings for (**c**) carbon, (**d**) Ni, (**e**) Co, and (**f**) Mn, with inset color scales illustrating atomic percentages. (**g**) Ion chromatograms depict the dissolved NCM cathode (standard, black line) and electrolytes post zero (red line), two (blue line), and 500 (pink line) cycles. The inset graph illustrates the full-range scaled chromatogram. Reprinted from Ref. [67] with permission from Elsevier, and (**h**) chronology of events leading to capacity loss: a reconstructed sequence. Reprinted from Ref. [68] with permission from ECS.

Surface electrode reactions with electrolytes are a significant factor contributing to capacity fading in Ni-rich cathode materials. Dissolution of transition metals, especially Ni and Mn, resulting from electrode–electrolyte interactions, affects both the cathode and anode materials in LIBs and eventually leads to electrochemical performance degradation by capacity fading and impedance increase [60,61,62,63,64]. S. J. Wachs et al. [65] conducted online monitoring to assess the dissolution of transition metals in NCM811. The researchers observed a significant increase in the concentration of transition metals in the electrolyte over time, particularly at high operating cutoff voltage. This phenomenon intensified with an extended duration of electrode polarization and through multiple charge-discharge cycles. A similar observation was made for NCM111, where the dissolution of transition metals, especially Mn, was detected at a high cutoff voltage [66]. D. Ko et al. [67] explored the correlation between cell impedance and the dissolution of transition metals in Li_1.0_Ni_0.87_Co_0.09_Mn_0.04_O_2_ cathode materials. Their investigation suggested that the hydrofluoric acid (HF), resulting from the interaction of a polyfluoroanion-based salt in the electrolyte with trace amounts of water or the breakdown of the organic electrolyte, ultimately contributed to the dissolution of transition metals. Consequently, the presence of H^+^ ions in the electrolyte instigated an attack on the electrode surface, leading to transition metal dissolution. This inference indirectly underscores that particles exhibiting greater microcracks are particularly prone to capacity deterioration, elucidating the adverse impact of microcrack generation and propagation on cathode materials. The backscattered electron image in Figure 9a illustrated the graphite surface after 500 cycles, with white areas indicating the deposition of elements heavier than carbon, such as transition metals. The electron probe microanalysis spectrum (Figure 9b) verified the presence of Ni on the anode surface. Notably, Co and Mn, due to their low concentrations, were undetected on the anode surface, contrasting with the clear presence of Ni (Figure 9c–f). The ion chromatograms graph in Figure 9g revealed that transition metals leaching from the cathode initiated from the second cycle reached concentrations of 2.56, 0.43, and 0.06 ppm for Ni, Co, and Mn, respectively. This leaching led to either deposition on the anode surface or the formation of transition-metal-containing polymerized organic matter in the electrolyte. J. A Gilbert et al. [68] suggested a model for mechanism cell impedance increase in NCM523/Gr where transition metals dissolve in the electrolyte and migrate to the SEI layer on the graphite, which changes its properties and traps more lithium. The explanation for the change in the SEI layer was provided, and Figure 9h can be helpful for better understanding. The capacity fading in high upper cutoff voltages is divided into three stages. In stage I, growth of SEI is a diffusion-controlled process in cutoff voltages lower than 4.3 V; however, when cutoff voltage increases above 4.30 V, a dramatic changeover occurs due to deterioration in an unpredictable, stochastic manner, and in the process, it loses a fraction of transition metals as ions. These ions subsequently migrate toward the negative electrode and become incorporated into SEI. After a relatively short induction period (stage I), deposition of these ions causes rapid growth of SEI with a rate that significantly exceeds the typical diffusion-controlled rates (stage II). Once this SEI grows sufficiently thick, a diffusion-controlled growth resumes at a new, higher rate (stage-III). In this state, further stress fracture of the oxide grains largely ceases due to the depletion of suitable sites. The alternative explanation is that oxide fracture continues, but it does not result in rapid SEI growth. The SEI coating becomes so thick that deposition of transition metals ions on its top does not result in more reduction of the material, as these deposited ions become separated from the reactive surface.

The safety and thermal abuse tolerance attributes of LIBs are serious concerns, particularly in EVs and large-scale applications. Within ternary Ni-based active material systems thermal stability is contingent upon the Ni concentration. At highly delithiated states, NCM and NCA cathode materials cause the reduction of Ni^4+^ during heating to release oxygen, initiating reactions with flammable electrolytes and leading to thermal runaway. Throughout the charging and overcharging cycling, the migration of Li^+^ ions from the bulk to the surface occurs, creating a concentration gradient between the core and the surface, resulting in non-uniform Li distribution within the particle. Determined by the lithium concentration in Li_x_MO_2_, the material decomposes into layered, spinel, and rock-salt structures, accompanied by the release of oxygen. The relationship between O_2_ release reactions and the extent of delithiation in Li_x_NiO_2_ can be understood by examining Reactions (1) and (2) [69,70]:

Li_x_NiO_2_ (layered) = (1 − x)LiNi_2_O_4_ (spinel) + (2x − 1)LiNiO_2_ (layered), 0.5 < x < 1, (R1)

Li_x_NiO_2_ (layered) = xLiNi_2_O_4_ (spinel) + (1 − 2x)NiO (rock salt) + ((1 − 2x)/2)O_2_, 0.5 > x (R2)

**Figure 10:**
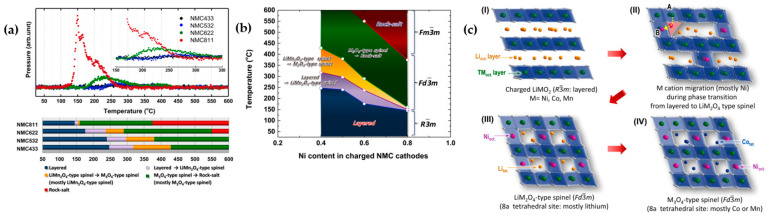
(**a**) Simultaneous collection of mass spectroscopy profiles for oxygen during TR-XRD measurements, along with the corresponding temperature region depicting phase transitions for NCM samples (lower panel), (**b**) an illustrative schematic depicting the phase stability map of charged NCM cathode materials during heating, and (**c**) schematic illustration depicting phase transition and potential migration paths of transition metal cations in charged NCM cathode materials during thermal decomposition: (**I**) in charged NCM cathode, TM and Li cations occupy octahedral and alternate octahedral sites, respectively, (**II**) phase transition from layered to spinel structure and migration of some TM cations to the Li octahedral sites, (**III**) displacement of Li ions from their original sites to the adjacent tetrahedral sites in first phase transition, and (**IV**) most of the octahedral sites are occupied by TM cations and M_3_O_4_-type spinel structure is reached. Reprinted from Ref. [71] with permission from the American Chemical Society.

Bak et al. [71] delineated a specific path of phase transitions correlated with oxygen evolution in charged Ni_x_Mn_y_Co_z_ (x:y:z = 4:3:3, 5:3:2, 6:2:2, and 8:1:1) as a function of heating temperature. The authors verified that phase transition in NCM cathodes went from a layered structure at room temperature to a disordered LiMn_2_O_4_-type spinel at temperatures above 150 °C (depending on NCM chemistry). A subsequent transition to a new spinel structure—assigned as a M_3_O_4_-type spinel (such as Co_3_O_4_ with Fd-3m space group)—and a rock-salt structure occurred at elevated temperatures. The primary distinction between the two spinel structures lay in cation occupations, particularly at the 8a tetrahedral sites. In the first spinel structure, Li occupies the 8a tetrahedral sites, while in the latter, transition metal cations migrate to these sites. Notably, the formation of the M_3_O_4_-type spinel delayed the formation of the rock-salt structure to a higher temperature, resulting in improved thermal stability [72]. As illustrated in Figure 10a, the phase transition to spinel structures occurred at lower temperatures, with a slight increase in Ni, indicating fewer transition metal migrations to 8a tetrahedral sites in NCM samples with less than 50% Ni. It is evident that the rock-salt structure formed at 365 °C and 550 °C in NCM811 and NCM622, respectively, while no rock-salt formation was observed for the other two samples up to 600 °C. Interestingly, O_2_ release aligned with phase transitions in all samples. Considering O_2_ release and phase transition, Figure 10b confirmed that in higher Ni content, the onset and range of temperature for O_2_ release were lower and narrower, respectively. Figure 10c explicitly elucidates the mechanism of phase transition in charged NCM during heating. Initially, transition metal cations occupied the octahedral layer, while Li^+^ ions were situated in alternate layers of octahedral sites. The first phase transition from layered to disordered occurred as transition metal ions migrated through favorable tetrahedral sites due to a lower energy barrier [73,74]. In this step, Li ions occupied tetrahedral sites, and a LiMn_2_O_4_-type spinel was formed. At high temperatures, a phase transition to a M_3_O_4_-type spinel structure transpired due to partial transition metal occupation in 8a tetrahedral sites. Due to the high stability and different electronic configurations of Co^2+^ and the instability of Ni^2+^ in tetrahedral sites, Ni ions preferentially occupied octahedral sites [75]. This explanation underscores the role of the Ni:Co:Mn molar ratio in the thermal stability of NCM cathodes. With lower Co concentration in NCM811, and consequently rapid Co cation migration to tetrahedral sites, the LiMn_2_O_4_-type spinel phase transition occurred rapidly at lower temperatures, and the temperature range for the thermally stable M_3_O_4_-type spinel type was narrower. It was suggested that NCM532 was the optimized sample from the thermal stability perspective and higher Mn content in the surface effectively increased thermal stability behavior of NCM materials. As explained earlier, the presence of NiO with a rock-salt structure at the cathode surface results in an increase in impedance during cycling, leading to degradation in the cycle capacity and thermal stability of the battery [71,76]. L. Wu et al. [76] conducted thermodynamic principle calculations based on oxidation and enthalpy reactions (Reaction (3) and Equation (1)) to elucidate the preferential formation of a rock-salt structure on the particle surface in Ni-rich cathodes:

2MO_2_ (layered) = 2MO (rock-salt) + O_2_, (R3)

ΔH = 2E (MO) + E(O_2_) + 2E (MO_2_) (1)

A negative enthalpy indicates that the decomposition of MO_2_ to rock-salt MO with the release of O_2_ is exothermic, signifying that the MO structure is more stable. In contrast, for positive enthalpy, MO_2_ is more stable than MO. The average reaction enthalpy is −0.8 eV and 1 eV per O_2_ for Ni_0.8_Co_0.2_O_2_ and Ni_1/3_Co_1/3_Mn_1/3_O_2_, respectively. This indicates that it is thermodynamically stable to form rock-salt in Ni-rich cathodes, and the MO structure is energetically favorable. It is noteworthy that the energy required to decompose MnO_2_, CoO_2_, and NiO_2_ to rock-salt is approximately 3 eV/O_2_, 1.7 eV/O_2_, and −1.5 eV/O_2_, respectively. These values clarify why Ni-enriched cathodes, such as LiNiO_2_, are thermally unstable compared to NCM111. Therefore, to enhance the thermal stability of Ni-based materials, it is suggested to increase Mn concentration at the surface of particles and Ni in the bulk.

**Figure 11:**
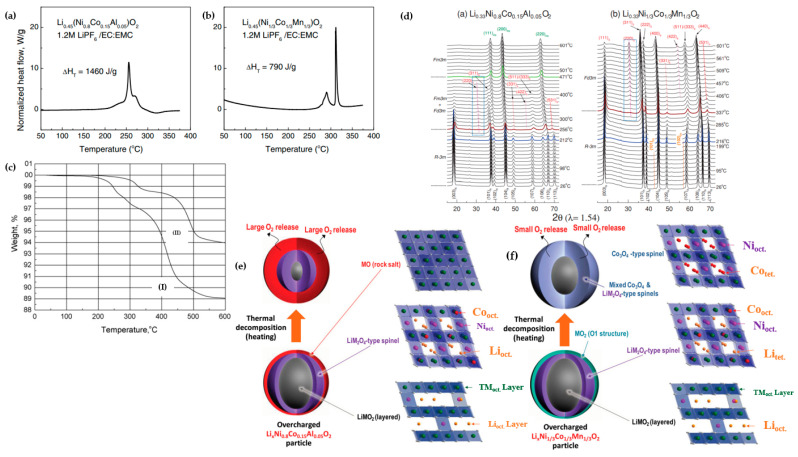
(**a**,**b**) DSC profiles illustrating the characteristics of Li_0.45_(Ni_0.8_Co_0.15_Al_0.05_)O_2_ and Li_0.55_(Ni_1/3_Co_1/3_Mn_1/3_)O_2_ powders, with a 1.2 M LiPF_6_/EC:EMC (3:7 wt%) electrolyte. Reprinted from Ref. [77] with permission from Elsevier (**c**) TGA under a purified air atmosphere reveals the delithiation behavior of (**a**) Li_0.45_(Ni_0.8_Co_0.15_Al_0.05_)O_2_ and (**b**) Li_0.55_(Ni_1/3_Co_1/3_Mn_1/3_)O_2_ cathodes. Reprinted from Ref. [78] with permission from Elsevier. (**d**) TR-XRD patterns captured during the heating process up to 600 °C for overcharged cathodes: (**I**) Li_0.33_(Ni_0.8_Co_0.15_A_l0.05_)O_2_ and (**II**) Li_0.33_(Ni_1/3_Co_1/3_Mn_1/3_)O_2_. The cathode samples, subjected to overcharging, were sealed in quartz capillaries, and heated from 25 to 600 °C for 4 h during the TR-XRD measurement, with a heating rate of 2.4 °C min^−^^1^, (**e**) structural changes during the thermal decomposition of overcharged Li_x_Ni_0.8_Co_0.15_Al_0.05_O_2_ cathode, presented schematically, and (**f**) illustration depicting the mechanism of thermal decomposition during heating in an overcharged Li_x_Ni_1/3_Co_1/3_Mn_1/3_O_2_ cathode. Reprinted from Ref. [72] with permission from John Wiley and Sons.

Structural transformations during the heat treatment, including R-3m → Fd-3m and Fd-3m → Fm-3m, were observed in delithiated NCM111 and Ni_0.8_Co_0.15_Al_0.05_, where O_2_ release and thermal decomposition occurred at a lower temperature for NCA compared to NCM111. Differential scanning calorimetry (DSC) showed exothermic peaks (Figure 11a,b) for both cathodes and electrolyte reaction, while NCM111 had higher onset temperature (beyond 300 °C) and lower heat generation, at 790 J g^−1^ compared to NCA (250 °C and 1460 J g^−1^) [77]. TGA substantiated the release of O_2_ at elevated temperatures for both cathodes, albeit with varying quantities. The thermogravimetric analysis (TGA) profiles (Figure 11c) depicted alterations in the weight of delithiated samples, indicating O_2_ loss from the lattice structure. The structural instability of NCA is evident, leading to O_2_ release at lower temperatures and a higher mass loss compared to NCM111—11% and 6% at 600 °C, respectively [78]. Time-resolved XRD (TR-XRD) is a powerful technique to afford researchers a better understanding of structural changes in active materials. TR-XRD patterns of overcharged NCM111 and Ni_0.8_Co_0.15_Al_0.05_ revealed that, in accordance with DSC and TGA profiles, the starting and completion points of phase transition are at 216 °C and 212 °C, and 337 °C and 256 °C, respectively. By heating up to 400 °C, another spinel phase was observed for NCA, while there was nothing for NCM111. As mentioned before, the formation of an M_3_O_4_-type spinel structure pushes formation of the rock-salt structure [72].

Organic carbonates, particularly ethylene carbonate (EC), and various linear carbonates, such as dimethyl carbonate (DMC), ethyl methyl carbonate (EMC), and diethyl carbonate (DEC), serve as the primary electrolyte components in LIBs [79]. The cathode surface of charged Ni-rich compositions exhibits a higher propensity for releasing reactive oxygen compared to other compositions, posing a significant safety risk. The released oxygen can potentially react violently with the flammable electrolytes within the battery, thereby risking a catastrophic explosion [80,81,82]. Furthermore, significant CO and CO_2_ gas formation ensues due to the decomposition of the electrolyte, reactions with impurities in the cell, or the release of gases trapped inside the particle. This phenomenon induces an increase in internal pressure, leading to mechanical stress within the electrodes and localized fractures. Notably, this occurrence was observed at lower cell potentials, approximately around 4.2 V (vs graphite), for NCM811, whereas it became apparent at 4.6 V for both NCM111 and 622 [83,84,85].

**Figure 12:**
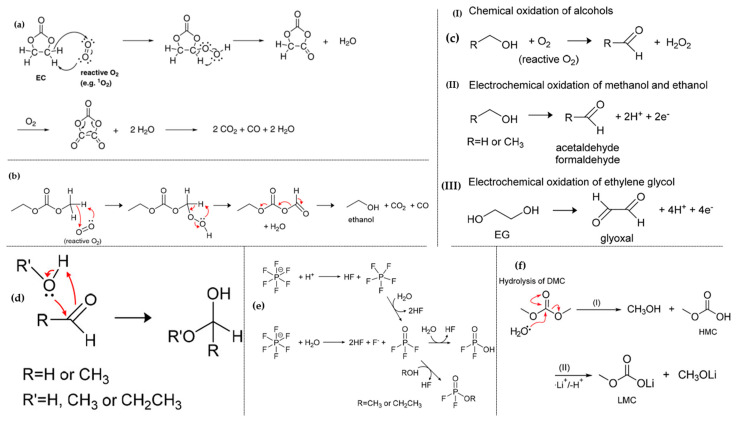
(**a**) Proposed mechanism for the oxidation of EC with reactive oxygen released from the NCM structure and yielding CO_2_, CO, and H_2_O. Reprinted from Ref. [54] with permission from ECS. (**b**) Hypothesized mechanism illustrating the chemical oxidation of EMC and the production of CO_2_, CO, and H_2_O, (**c**) comparative analysis of chemical and electrochemical oxidation routes for the conversion of alcohols to aldehydes, (**d**) illustration of nucleophilic attack on aldehydes by water or alcohols, resulting in the formation of acetals, (**e**) visualization of the decomposition process of LiPF_6_ salt. Reprinted from Ref. [87] with permission from American Chemical Society. (**f**) Proposed reaction scheme depicting the chemical oxidation of DMC leading to the formation of formic acid, CO_2_, and Water. Reprinted from Ref. [90] with permission from the American Chemical Society.

Upon lithium extraction in NCM materials, the c-parameter increases until roughly 2/3 of the lithium is removed. This increase is attributed to repulsive interactions of negatively charged oxygen layers upon the removal of positive Li ions. O_2_ evolution from NCM cathode materials correlates with the SOC (Li removal), indicating an increased instability at high degrees of delithiation. Generally, O_2_ evolution initiates at a critical SOC of about 80% for all layered active materials, irrespective of the potential at which this SOC is reached. However, Ni-rich materials reach the critical SOC at lower potentials due to their flatter potential profile. Subsequent Li removal at higher SOC results in a decreasing c-parameter, linked to increasing covalency between the metal and oxygen. This covalency corresponds to a decrease in the oxygen anion charge density, exemplified by the oxidation of lattice oxygen anions. Upon reaching the critical SOC of about 80%, the evolution of singlet oxygen is a chemical reaction of the electrode material, directly or indirectly caused by Li deficiency, leading to the loss of lattice oxygen as enough lithium is removed to destabilize the crystal structure. The H2 → H3 phase transition occurred in high levels of SOC at about 4.2 V; Li/Li^+^ is hypothesized to contribute to the shrinkage of the c-parameter for NCM811, and this phenomenon could result from decreasing repulsion between the oxygen layers caused by the oxidation of oxygen anions, ultimately leading to O_2_ release [54,86]. The O_2_ release is the catalyst for the generation of CO and CO_2_ within battery cells. CO_2_, a widely recognized byproduct, arises from the irreversible oxidation of carbonate solvents employed in battery electrolytes. Illustrated in Figure 12a, the potential for EC oxidation primarily resides in the two carbon atoms bound to hydrogen, as the carbonyl-carbon is already in its maximum oxidation state. The underlying mechanism initiates an electrophilic attack on the carbon by the O_2_ molecule, leading to the formation of a peroxo group carrying the proton initially bound to the carbon. This relatively unstable peroxo group undergoes immediate decomposition, resulting in the formation of a carbonyl group and the release of a water molecule. Subsequent decomposition of this molecule has the potential to yield CO, CO_2_, and formaldehyde. Reaction (4) encapsulating this process is represented as follows [54]:

EC + 2O_2_ (lattice) = 2CO_2_ + CO + 2H_2_O (R4)

O_2_ release is responsible for the generation of CO and CO_2_ in battery cells due to reaction with other organic carbonates. CO and CO_2_ gassing with a reduced ratio compared to EC were observed in the reaction of EMC and reactive oxygen release from NCM811 by W. M. Dose et al. [87]. EMC decomposition pathways to ethanol, CO_2_, and CO were proposed, considering its chemical and electrochemical instability in the presence of reactive lattice oxygen [87]. The proposed pathways for EMC decomposition to ethanol and the production of CO_2_ and CO are illustrated in Figure 12b, and is represented as Reaction (5). Considering the chemical and electrochemical instability of ethanol in the presence of reactive lattice oxygen, multiple pathways for oxidation of ethanol to acetaldehyde and either peroxide or protons are possible, as shown in Figure 12c [87].

EMC + O_2_ (lattice) = EtOH + CO_2_ + CO + H_2_O (R5)

Furthermore, water produced by the decomposition of EC and EMC contributes to further degradation. The reaction of nucleophilic attack by water with aldehydes may lead to acetal formation (Figure 12d). Additionally, LiPF_6_ decomposition is more likely in the concomitant presence of water and protons, leading to capacity fading and promoting higher concentrations of acidity and proticity that can migrate to the negative surface and degrade the solid electrolyte interface layer (Figure 12e) [88]. DMC is highly prone to hydrolyzation compared to EC. The intermediate products of DMC hydrolysis, formic acid and lithium methyl carbonate (LMC), undergo further chemical oxidation, forming formic acid, water, and CO_2_ (Figure 12f) [89,90].

Excess Li is typically used in Ni-rich cathode materials to decrease Li/Ni cation mixing, aiding in obtaining a well-ordered layer structure. However, Li_2_O remains at the surface, and LiOH and Li_2_CO_3_ impurities form easily during preparation and storage due to the slow and spontaneous reduction of Ni^3+^ to Ni^2+^. The presence of residual lithium compounds is unfavorable, as they cause unwanted side reactions through oxidative decomposition of LiOH and Li_2_CO_3_ at high voltages, resulting in irreversible capacity. The formation of Li_2_CO_3_ is presumed to take place via Reaction (6). In the case of Li(NiCoAl)O_2_ electrode materials, these compounds are prone to reaction with CO_2_ and moisture in the air [91,92,93,94,95].

Li(NiCoAl)O_2_ + 1/4xO_2_ + 1/2xCO_2_ = Li_1−x_(NiCoAl)O_2_ + 1/2xLi_2_CO_3_ (R6)

The performance of active materials deteriorates, as LiOH increases the pH during electrode processing, causing gelation [96]. Additionally, Li_2_CO_3_ causes gas generation and cell swelling, potentially leading to safety issues in LIBs [97]. In a worse scenario, acidic HF forms as a result of the reaction between LiOH and LiPF_6_ in the electrolyte. This leads to transition metal dissolution at the charged state, water and CO_2_ gas evolution, formation of an insulating surface layer, and phase transition to a spinel structure [92,98,99,100]. CO_2_ outgassing by Li_2_CO_3_ decomposition may occur through possible pathways, including an electrochemical process in which Li_2_CO_3_ can decompose near its standard potential, E° = 3.82 V vs. Li/Li^+^, with Reaction (7). Although Kaufman et al. [101] claimed that that the electrochemical decomposition of Li_2_CO_3_ did not necessarily generate oxygen gas, the production of a reactive oxygen species is possible, and these species can directly or indirectly participate in electrolyte degradation processes [102,103].

2Li_2_CO_3_ = 4Li^+^ + 4e^−^ + 2CO_2_ + O_2_ (R7)

**Figure 13:**
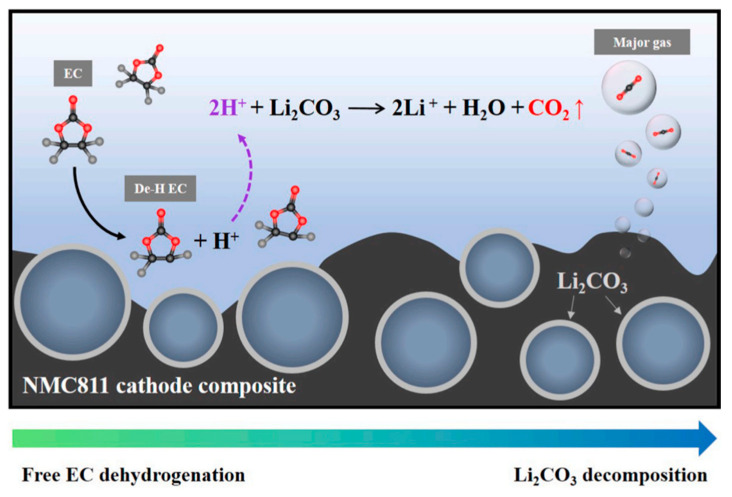
Proposed mechanism detailing the chemical decomposition of Li_2_CO_3_ triggered by the dehydrogenation of free EC within an ethylene carbonate-based electrolyte. Reprinted from Ref. [104] with permission from Elsevier.

It is also suggested that an increase in free EC concentration near the oxide cathode surface, easily adsorbed due to its higher dielectric constant compared to commonly used linear alkyl carbonate, facilitates the decomposition of Li_2_CO_3_ (Reactions (8) and (9)), as illustrated in Figure 13 [104].

EC (free) = De–HEC + H^+^ + e^−^, (R8)

Li_2_CO_3_ + 2H^+^ = 2Li^+^ + H_2_O(l) + CO_2_(g) (R9)

## 3. Strategies and Countermeasures

Extensive research has been undertaken to enhance Ni-rich cathode materials performance in response to the growing demand for batteries with higher energy density. Common strategies employed for this purpose include elemental substitution, surface treatment, compositional adjustments, as well as the utilization of single crystals and concentration gradient structures. These methods are widely adopted to address the associated challenges and requirements effectively.

### 3.1. Elemental Doping

**Figure 14:**
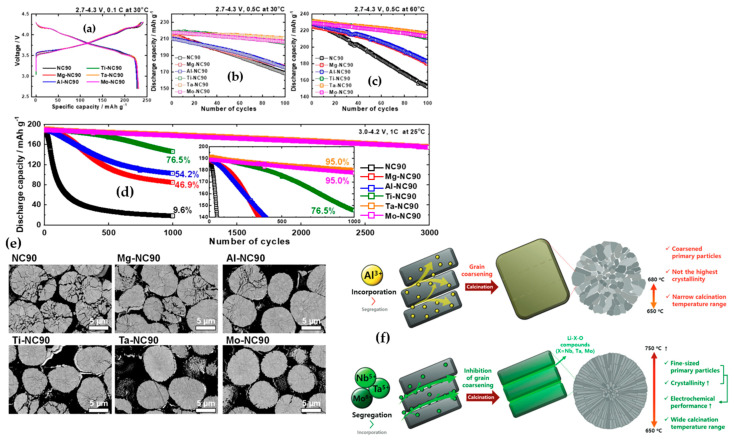
Performance evaluation of cathodes (NC90, Mg-NC90, Al-NC90, Ti-NC90, Ta-NC90, and Mo-NC90) in pouch-type full cells, (**a**) showcasing first charge–discharge cycle curves at 0.1C and 30 °C, (**b**,**c**) cycling at 0.5C over 100 cycles at both 30 °C and 60 °C, and (**d**) extended cycling at 1C and 25 °C within the voltage range of 3.0–4.2 V vs. graphite; (**e**) presents cross-sectional images of recovered cathodes after 1000 cycles at 100% DOD, discharged state of 2.7 V. Reprinted from Ref. [134] with permission from Springer Nature. (**f**) Distinctions in the mechanisms and impacts of low-valence (Al) and high-valence (Nb, Ta, and Mo) dopants, encompassing their incorporation into the bulk material and the coating of grain boundaries. Reprinted from Ref. [135] with permission from John Wiley and Sons.

The incorporation of cation and anion doping has emerged as a prominent strategy to enhance the structural stability of Ni-enriched layered oxides [105,106]. This approach proves pivotal in improving the cycling performance of cathode materials by substituting unstable elements with inactive materials. It effectively hinders microcrack propagation within primary particles through the pillaring effect of cationic species, while stabilizing the oxygen octahedral site through anionic doping [107,108]. As an example, Mg emerged as a promising cation element with the potential to serve as an inert stabilizer, leading to enhanced performance in NCM622 cathode materials by mitigating structural deterioration. The introduction of Mg at concentrations of 1%, 3%, and 5% resulted in a reduction of Li/Ni cation intermixing by 1.58%, 1.77%, and 3.20%, respectively, compared to the 5.18% observed in undoped NCM. When Mg ions partially substituted for Ni in Mg-doped NCM, the unit cell parameters a and c decreased in magnitude, while the c/a ratios increased. This change typically signified the development of a layered material structure. The reduction in unit cell dimensions a and c was attributed to the incorporation of Mg^2+^ ions into both the Li layer and the transition metal layer. This incorporation led to a decrease in the overall content of Ni^2+^ ions residing within the Li layer. This phenomenon is comprehensible because the diminished presence of Ni^2+^ ions in the structure of Mg-doped powders resulted in a slight reduction in lattice parameters. Furthermore, it is noteworthy that the bond dissociation energy of Mg-O (∆H_f298_ = 394 kJ mol^−1^) surpasses that of Ni-O (∆H_f298_ = 391 kJ mol^−1^). Therefore, the introduction of Mg^2+^ ions into the host structure contributed to the enhancement of structural stability [109]. Anionic dopants, like F, have the capacity to occupy oxygen sites without compromising the capacity and energy density of the active materials. Among these anionic dopants, fluorine exhibits the highest electronegativity and establishes a remarkably stable structure due to the formidable bonds formed between transition metals and fluorine. Furthermore, the enhanced electrochemical performance of cathode materials doped with fluorine may be attributed to their shielding effect against HF production, which can arise during the decomposition of the liquid electrolyte [110,111,112]. Sung-Beom Kim et al. [113] employed a solid-state reaction method to synthesize F-doped NCM811. The pristine material exhibited a discharge capacity of 97.9 mAh g^−1^ after 100 cycles at a rate of 100 mA g^−1^, within the voltage range of 2.8–4.3 V. In contrast, the discharge capacities of LiNi_0.8_Co_0.1_Mn_0.1_O_2−x_F_x_, with x values of 0.02, 0.04, 0.06, and 0.08, were measured at 107.8, 133.2, 169.6, and 154.5 mAh g^−1^, respectively. Furthermore, assessing the capacity retention of various samples, including NCM-bare, NCMF-2, NCMF-4, NCMF-6, and NCMF-8 after 100 cycles, revealed retention percentages of 60.7%, 66.2%, 78.8%, 96.8%, and 89.5%, respectively. A diverse array of elements has been successfully employed as cation and anion dopants for transition metals and oxygen sites, aiming to enhance the electrochemical performance of Ni-rich cathode materials. Notable examples include Mo [114,115], Al [116,117], B [118], Nb [119,120,121], K [122], Na [123], Mg [109], Ti [124,125,126,127,128,129,130], PO_4_^3-^ [131], and F [113,132,133]. It is worth mentioning that doping Ni-rich cathode materials with cations of varying valence states engenders distinct mechanisms that contribute to performance enhancement. Figure 14a–d provides a visual representation of the electrochemical effects resulting from a 1% molar elemental doping with diverse valence states on LiNi_0.91_Co_0.09_O_2_ active material [134]. Notably, all doped materials, irrespective of low valence (Mg^2+^ and Al^3+^) or high valence (Ti^4+^, Ta^5+^, and Mo^6+^) states, exhibited superior electrochemical performance compared to the undoped counterpart in initial cycles, regardless of cycling rate and temperature. While samples doped with low valence states displayed satisfactory performance over extended cycles, such as 1000 cycles, capacity retention significantly decreased to 54.2% and 46.9% for Al and Mg-doped samples, respectively. In contrast, Ta- and Mo-doped samples maintained capacity retention even after 3000 cycles. Structural evaluations of samples, depicted in cross-sectional SEM images (Figure 14e) of cathodes cycled up to 1000 cycles, revealed that undoped NC90 exhibited severe intergranular cracks and disintegration into individual grains. Conversely, Mg and Al-doped samples showed incipient microcrack propagation, while Ta and Mo-doped samples retained particle coherency with minimal signs of microcrack nucleation and propagation, aligning with their exceptional cycling stabilities. These observations suggest diverse mechanisms contributing to the enhancement of doped samples.

Park et al. [135] have proposed mechanisms for improvement through cation doping with different valence states (Figure 14f). The authors investigated the effects of a 1% molar doping of Al^3+^, Nb^5+^, Ta^5+^, and Mo^6+^ in LiNiO_2_. They suggested that cations with low valence states, such as Al^3+^, participated in the bulk and acted as pillars to improve structural stability and electrochemical performance. Regarding high valence cations, such as Mo^6+^, Nb^5+^, and Ta^5+^, these elements not only participated in the bulk structure, but also reacted with Li resources and Li-X-O compounds forming and segregating at grain boundaries, inhibiting grain boundary migration, and preserving the size of primary particles. For instance, Al 1% molar doping in LiNiO_2_ resulted in LiAlO_2_ formation at 650 °C, disappearing at 680 °C. In contrast, compounds such as LiNbO_3_ appeared at low temperatures, and at higher calcination temperatures, the insoluble Li_3_NbO_4_ phase were segregated in the grain boundaries. Consequently, if doping elements persisted as Li-X-O compounds during high-temperature calcination, they coated the grain boundary, inhibiting boundary migration and suppressing primary particle coarsening. For elements like Al and Mg with low valence states, it was suggested that higher doping molar concentrations (3%) could be beneficial for grain boundary reinforcement. In a study by Hüger et al. [136], it was revealed that compounds like LiNbO_3_ and LiTaO_3_ exhibit higher Li diffusivity, or in other words, had a higher Li insertion/extraction rate (about 1 × 10^−18^ and 8 × 10^−19^ m^2^ s^−1^, respectively) compared to other compounds such as LiAlO_2_ and LiGaO_2_ (about 4 × 10^−21^ and 1 × 10^−21^ m^2^ s^−1^, respectively). Furthermore, the bond dissociation energy of Nb-O and Ta-O is nearly twice (∆H_f298_ = 753 and 805 kJ mol^−1^, respectively) that of Mg-O and Ni-O, thereby enhancing structural stability. The published works in recent years on the doping of Ni-rich cathode materials are summarized in Table 3.

### 3.2. Surface Coating

**Figure 15:**
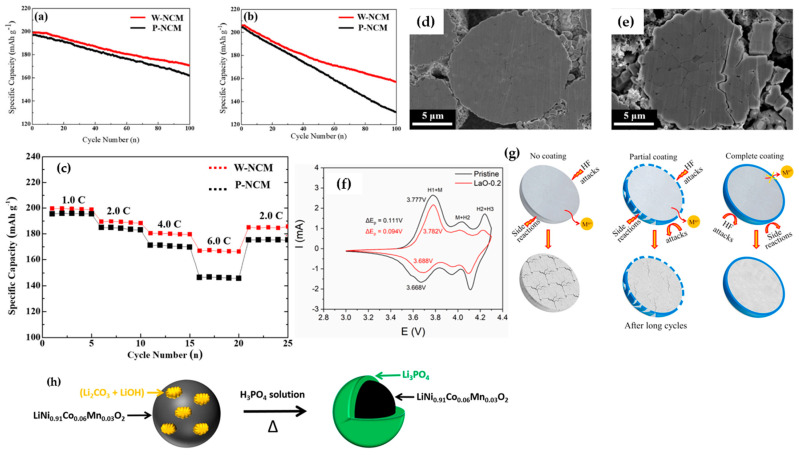
Cyclabilities of pristine and WO_3_-coated NCM at a voltage of (**a**) 3.0–4.3 V and (**b**) 3.0–4.6 V at 0.5C, (**c**) rate capabilities of pristine and WO_3_-coated NCM. FE-SEM images after 100 cycling for (**d**) WO_3_ coated NCM and (**e**) pristine NCM. Reprinted from Ref. [165] with permission from Elsevier. (**f**) Cyclic voltammetry curves for pristine and LaO-0.2. Reprinted from Ref. [166] with permission from Elsevier. (**g**) Mechanism for the protective action of TiO_2_ nano-coating on Ni-rich cathode materials. Reprinted from Ref. [167] with permission from Elsevier. (**h**) Schematic illustration depicting the transformation of residual lithium into a Li_3_PO_4_ coating. Reprinted from Ref. [175] with permission from Springer Nature.

Surface treatment represents a foremost strategy to mitigate interfacial side reactions between cathode materials surface and electrolytes, with the aim of enhancing the retention of oxide ion vacancies within the crystal lattice post-initial charge. This approach concurrently addresses challenges such as the suppression of electrolyte decomposition, the mitigation of cathode-electrolyte interphase formation, the preservation of low microstrain for improved structural integrity and crystallinity during cycling, and the sequestration of HF from the electrolyte [154,155,156,157,158]. In the context of Ni-rich materials, a coating which is generally composed of nanoparticles and typically ranging from 5–20 nm can be broadly classified into three categories: oxides, phosphates, and fluorides. Among these, metal oxide coatings, including Al_2_O_3_ [159], Li_4_Ti_5_O_12_ [160], ZrO_2_ [161,162], Li_2_WO_4_ [163], and Li_2_ZrO_3_ [164] have emerged as widely adopted choices to address the limitations of Ni-rich materials. Shim et al. [165] specifically applied an acidic WO_3_ coating to NCM811 particles, leveraging its intercalation host properties attributed to its well-defined cubic ReO_3_-type structure and high resistance against HF attack. As depicted in Figure 15a,b, the WO_3_ coating significantly enhanced the cycling performance of NCM811 within the 3–4.3 V range, and notably, under challenging conditions spanning a voltage range of 3–4.6 V. The capacity retention of the pristine active material at upper cutoff voltages of 4.3 V and 4.6V was 82% and 64.44%, respectively, while for the WO_3_-coated NCM811, the corresponding values were 85.8% and 76.46%, respectively. In terms of rate capability performance (Figure 15c), the coated sample exhibited superior performance attributed to expedited Li ion and electron kinetics, stemming from enhanced structural stability and reduced resistance. This improvement was further attributed to the suppression of side reactions between the cathode material and electrolyte. Moreover, Figure 15d,e illustrates the absence of discernible microcracks along grain boundaries in NCM protected by a 2.14 nm thick WO_3_ coating. In contrast, the untreated NCM, subjected to cycling-induced expansion and shrinkage, exhibited electrolyte infiltration into the interior, leading to structural collapse. This observation underscored the effectiveness of the WO_3_ coating in preserving the structural integrity of NCM during cycling. The incorporation of a La_2_O_3_ coating as a protective layer has proven highly effective in augmenting the electrochemical performance of the Ni_0.91_Co_0.06_Mn_0.03_ electrode in LIB [166]. Notably, the capacity retention of the cathode after 100 cycles at 0.5C demonstrated a substantial improvement, with a retention rate of 68.1% for the pristine NCM, whereas the La_2_O_3_-coated material exhibited superior retention at 87.2%. The cyclic voltammetry plots (Figure 15f) of both pristine and coated samples revealed significant improvements by coating deposition on NCM material. First, the intensity peak related to phase transformation H2 → H3 that is in charge of particle pulverization and capacity fading decreased significantly. Secondly, the oxidation-reduction gap (ΔE = E_oxidation_ − E_reduction_), which serves as a determinant of electrode polarization, reduced from 0.111 V to 0.094 V [165]. A TiO_2_ coating was employed to provide comprehensive protection for NCM811 cathode active materials, capitalizing on its structural stability and chemical inertness against the electrolyte at high voltages [167]. Q. Fan et al. [167] reported that the application of a continuous TiO_2_ nano-coating layer on NCM811 not only resulted in a higher specific discharge capacity, but also significantly improved cycling performance, with 72.2% capacity retention for the coated sample and 45.7% for the bare sample. This improvement was attributed to the reduction in side reactions at the electrode and electrolyte interface, particularly at high upper cut-off voltages. The exceptional barrier properties of the TiO_2_ coating led to a reduction in electrolyte consumption and the formation of a thick passivation solid electrolyte film. This reduction helped alleviate polarization at the material interface, preventing the inevitable damage resulting from direct contact between the active material and the electrolyte. Figure 15g illustrated a schematic representation of the protective mechanism of TiO_2_, emphasizing the significance of an intact coating for active materials. This depiction underscores that the development of a partial coating may result in the isolation failure of particles, potentially leading to the collapse of secondary particles. Phosphate coatings have emerged as highly protective coatings for matrix materials, acclaimed for their exceptional thermal and electrochemical stability. This stability arises from the robust P=O bond, with a bond energy of 5.64 eV and the formation of strong covalent bonds between polyanions and metal ions, rendering them resistant to chemical attacks [168,169,170,171]. Among phosphate coatings, the Li_3_PO_4_ coating stands out as a prominent choice for Ni-rich active materials due to its rapid Li^+^ conductivity and high chemical stability [172]. The wet-coating method, utilizing an aqueous solution of NH_4_H_2_PO_4_ or phosphoric acid, has been widely employed to extensively create Li_3_PO_4_ coatings on active materials, enabling a substantial reduction of lithium-based surface impurities (LiOH and Li_2_CO_3_) and the suppression of detrimental side reactions during both storage and cycling [173,174]. T. Sattar et al. [175] coated Li_3_PO_4_ onto layered oxide Ni_0.91_Co_0.06_Mn_0.03_ active material by transforming Li residual compounds into the coating (Figure 15h). The superior electrochemical rating and cycling performance of the coated samples were attributed to the removal of lithium residual compounds and the isolation of particles, preventing direct contact with the electrolyte and excessive growth of the solid electrolyte interface layer. Additionally, the higher dissociation energy bond of P=O compared to transition metal–oxygen bonds contributed to better electrochemical performance, with capacity retention rates of 68.1% for pristine NCM and 82.4% for the surface-modified sample. In the context of EV industries, where thermal stability is crucial, Ni_0.8_Co_0.15_Al_0.05_ cathode materials, employed in Tesla’s Model S and X, have gained prominence [176]. A Li_3_PO_4_ coating developed on Ni_0.815_Co_0.15_Al_0.035_ active material has been shown to decrease heat generation during cycling. A study conducted by Tang et al. [177] confirmed through DSC profiles that the Li_3_PO_4_-modified samples generated less heat (525 J g^−1^) compared to the pristine sample (757 J g^−1^), highlighting the efficacy of the coating in suppressing surface side reactions with the organic electrolyte. The entire process of reaction between Li residual compounds and phosphoric acid can be expressed through the following Reactions (10)–(12) [178,179]:

3Li_2_O + 2H_3_PO_4_ = 2Li_3_PO_4_ + 3H_2_O, (R10)

3LiOH + H_3_PO_4_ = Li_3_PO_4_ + 3H_2_O, (R11)

3Li_2_CO_3_ + 2H_3_PO_4_ = 2Li_3_PO_4_ + 3H_2_O + 3CO_2_ (R12)

Fluorides exhibit lower Gibbs energy compared to oxides, indicating their heightened stability in comparison to oxide counterparts. The utilization of metal fluorides as protective coatings has been extensively investigated to address challenges associated with electrode side parasitic reactions with the electrolyte and HF etching. Among the various fluorine coatings, LiF is frequently employed for Ni-rich active materials. A thermodynamically stable lithium fluoride coating, characterized by superior Li ion conductivity compared to other fluorides, has been typically achieved through the mixing of NH_4_F and NH_4_HF_2_ with the original cathode materials [180,181]. H. Kim et al. [182] treated Ni_0.85_Co_0.12_Al_0.03_ cathode material with NH_4_HF_2_ and reported that this treatment helped to remove Li residual compounds from the particles’ surface and additionally, both cycling performance and gas evolution behavior were enhanced significantly in the modified sample.

The published works in recent years on the coating development of Ni-rich cathode materials are summarized in Table 4.

### 3.3. Structural Engineering

**Figure 16:**
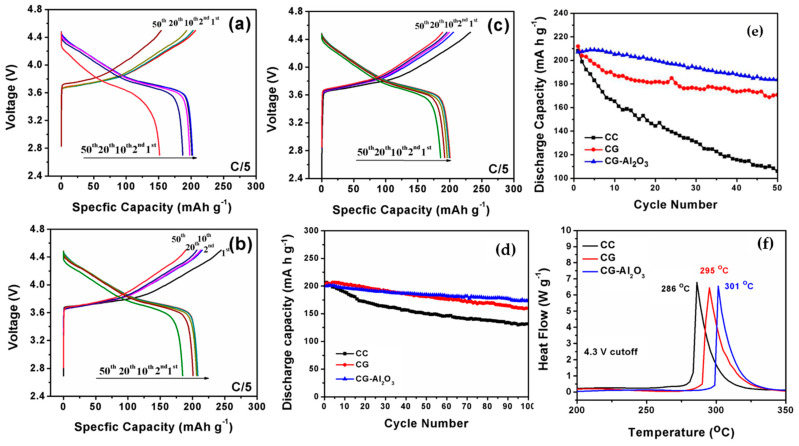
Charge discharge curves for (**a**) normal, (**b**) CG, and (**c**) CG-Al_2_O_3_ electrodes within the voltage range of 2.7 to 4.5 V at room temperature, comparative cycling performances (C/5) for the three cathodes at (**d**) room temperature and (**e**) 55 °C, and (**f**) DSC traces illustrating the heat flow during the reaction of the electrolyte with CC, CG, and CG-Al_2_O_3_ electrodes when charged to 4 V. Reprinted from Ref. [202] with permission from Elsevier.

**Figure 17:**
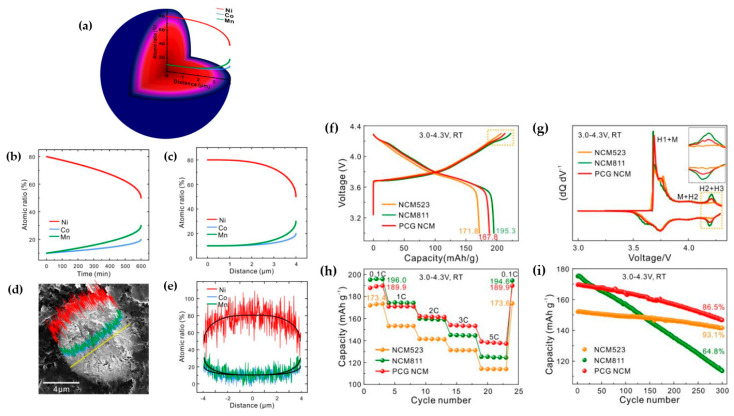
(**a**) Illustration depicting the distribution of transition metals within the concentration CG NCM particle, (**b**) profiles showing the calculated transition metal compositions over time, and (**c**) from the particle’s core to its surface. (**d**) EDS line scanning analysis conducted across the precursor particle (the yellow line denotes the scanning path), (**e**) comparative analysis of the measured composition profiles with the calculated outcomes. Electrochemical performance of CG NCM, NCM523, and NCM811 cells within the voltage range of 3.0–4.3 V: (**f**) initial charge–discharge profiles at a 0.1C rate, (**g**) differential capacity curves for the initial charge–discharge cycle, (**h**) rate capability assessed at different charge-discharge current densities, and (**i**) cycling performance evaluated at a 1C rate. Reprinted from Ref. [204] with permission from the Royal Society of Chemistry.

The innovative approach to developing functional layered oxide materials involves creating a comprehensive concentration gradient structure. This strategy utilizes a high-capacity Ni-rich core in conjunction with a chemically stable Co, Mn-rich surface to prevent undesired Ni^4+^ reduction on the material’s surface. In a typical cathode with a full concentration gradient, the concentration of Ni gradually decreases from the particle’s center to its outer surface, while the concentrations of Co and Mn increase [198]. Hou et al. [199] synthesized a full concentration gradient (FCG) NCM622 with LiNi_0.8_Co_0.2_O_2_ for the inner composition, and LiNi_0.4_Co_0.2_Mn_0.4_O_2_ for the outer composition. Reports indicated that the FCG-NCM622 cathode exhibited superior capacity retention at both room temperature and 55 °C. Moreover, DSC analysis confirmed the enhanced thermal stability of the structurally modified NCM622 compared to the normal sample. The exothermic peak observed at 282.7 °C for FCG-NCM622 and 266.2 °C for NCM622 indicated better thermal stability for the former. The generated heat was 692 J g^−1^ for FCG-NCM622 and 969 J g^−1^ for NCM622, attributed to the presence of Mn on the outer surface of the active material. Recently, a combination of FCG and coating-doping modifications has been employed to improve the thermal and electrochemical performance of Ni-enriched cathode materials [200,201]. Liao et al. [202] utilized dual modification for NCM material, incorporating both a concentration gradient and a protective Al_2_O_3_ coating. Figure 16a–c demonstrates that this dual modification significantly enhanced electrochemical performance, resulting in lower voltage decay over cycling compared to a normal cathode. This improvement was attributed to the reduced reaction of the cathode material with the electrolyte, and higher structural stability. The capacity retention for coated CG-NCM after 100 cycles was approximately 88%, outperforming CG (80%) and normal NCM (62.6%) as shown in Figure 16d. The impact of dual modification became more evident in cycling at higher temperatures (55 °C), as illustrated in Figure 16e. In terms of thermal stability, DSC profiles (Figure 16f) confirmed that both coated CG and CG samples exhibit a higher onset temperature for the exothermic reaction compared to the normal sample, indicating the effectiveness of both modifications. Park et al. [203] explored the electrochemical and thermal characteristics of a concentration gradient Ni_0.865_Co_0.120_Al_0.015_ cathode material. Although conventional NCA demonstrated a slightly higher initial discharge capacity at 0.1C in the operating potential range of 2.7–4.3 V compared to CG NCA (225 and 222 mAh g^−1^, respectively), the capacity retention of the CG electrode at 0.5C, both at room temperature and 60 °C, was notably superior to that of the standard sample. Moreover, differential scanning calorimetry (DSC) measurements on charged samples at 4.3 V revealed an exothermic peak at 192 °C with a heat generation of 1789 J g^−1^ for conventionally synthesized NCA, whereas for CG NCA, it was observed at 202 °C with a lower heat generation of 1409 J g^−1^, indicating enhanced thermal stability of the structurally modified NCA. Xu et al. [204] successfully synthesized a concentration gradient cathode material comprising Ni_0.7_Co_0.13_Mn_0.17_ with NCM811 composition at the core and NCM523 at the outer surface (Figure 17a–e). The initial charge–discharge profile (Figure 17f) of NCM523, NCM811, and the CG NCM indicated that the CG sample exhibited a lower discharge capacity compared to NCM811, attributed to its lower Ni content on the surface. Additionally, the plateau at around 4.2 V was shorter for NCM523 and CG compared to NCM811. Moreover, the H2 → H3 phase transition, recognized as the primary reason for volume change and subsequent capacity fading in Ni-rich electrodes, was pronounced for NCM811 compared to the CG cathode material (Figure 17g). The rate capability of the CG electrode outperformed that of NCM811 and NCM523 counterparts, delivering much higher capacity at high discharge rates (Figure 17h). Cycling profiles of the samples at 1C (Figure 17i) revealed that NCM811 experienced catastrophic capacity retention, while the performance of CG was highly similar to that of NCM523. These results suggest that utilizing the CG cathode offers benefits for both NCM523 in terms of capacity retention, and for NCM811 in terms of high capacity delivery.

## 4. Conclusions and Perspectives

Lithium-ion batteries are indispensable for meeting the surging demand for renewable energy solutions, offering high energy density, extended cycle life, and safety. Among materials for cathode in LIBs, Ni-enriched cathode materials stand out for their remarkable capacity and energy density, positioning them as promising contenders for future battery applications.

This review delineates the key hurdles hindering the widespread integration of Ni-rich cathode materials in electric vehicles (EVs). Structural instability and side reactions pose significant challenges, categorized into phase transitions, microcrack formation, cation mixing, and gas release. Various strategies, including elemental doping and surface coatings, are explored for enhancing battery lifespan and safety. Notably, the concentration gradient method emerges as a novel approach to bolster the performance of Ni-rich electrodes. However, further research is imperative, particularly in comprehending capacity fading mechanisms and optimizing modification techniques. Continuous investigation is essential to propel Ni-rich cathode materials towards widespread adoption in EVs and large-scale energy storage systems.

## Figures and Tables

**Table 1:** Primary criteria and obstacles encountered in EVs battery development. Reprinted from Ref. [10] with permission from Elsevier.

Battery Attributes	Main Requirements	Main Challenges
Energy density	>750 Wh L^−1^ and >350 Wh kg^−1^ for cells	Difficult to find one battery technology that meets all aspects; Tradeoffs must be managed effectively.
Cost	<$100 per kWh for cells	
Fast charge and power	80% ∆SOC * in 15 min	
Life	15 years	
Performance	Minimum impact by environment	
Safety	No fire/flame/rupture/explosion	
Temperature range	−30 to 52 °C [11]	

* SOC stands for State of Charge.

**Table 2:** I(003)/I(104) ratio for Ni_x_Co_y_Mn_z_ cathode materials with varying Ni content (x = 1/3, 0.5, 0.6, 0.7, 0.8 and 0.85). Reprinted from Ref. [48] with permission from Elsevier.

Layered Oxide Cathode Material Composition	I(003)/I(104)
Li[Ni_1/3_Co_1/3_Mn_1/3_]O_2_	1.35
Li[Ni_0.5_Co_0.2_Mn_0.3_]O_2_	1.32
Li[Ni_0.6_Co_0.2_Mn_0.2_]O_2_	1.26
Li[Ni_0.7_Co_0.15_Mn_0.15_]O_2_	1.20
Li[Ni_0.8_Co_0.1_Mn_0.1_]O_2_	1.19
Li[Ni_0.85_Co_0.075_Mn_0.075_]O_2_	1.18

**Table 3:** Electrochemical performance of Ni-rich cathode materials before and after doping.

Researchers	Doping Element	Composition	Testing Conditions	Pristine Material [mAh g^−1^]	Doped Material [mAh g^−1^]
Kim et al. [137]	Al	Ni_0.92_Co_0.04_Mn_0.04_	2.7–4.3 V RT * 0.5C	215 (initial) 174 (100 cycles)	202 (initial) 187 (100 cycles)
Do et al. [138]	Al	Ni_0.8_Co_0.1_Mn_0.1_	3.0–4.3 V RT 1C	172.4 (initial) 120 (100 cycles)	171.71 (initial) 163 (100 cycles)
Chu et al. [119]	Nb	Ni_0.8_Co_0.1_Mn_0.1_	2.7–4.5 V RT 2C	163.5 (initial) 97.3 (200 cycles)	202.8 (initial) 164.1 (200 cycles)
Zhang et al. [139]	Ti	Ni_0.8_Co_0.1_Mn_0.1_	2.8–4.3 V RT 1C	147.41 (initial) 96.23 (150 cycles)	165.6 (initial) 127.53 (150 cycles)
Wu et al. [140]	Ga	Ni_0.8_Co_0.1_Mn_0.1_	2.8–4.5 V RT 1C	205 (initial) 130 (100 cycles)	183 (initial) 174 (100 cycles)
Lu et al. [141]	Cu	Ni_0.6_Co_0.2_Mn_0.2_	2.7–4.4 V RT 5C	130.5 (initial) 80.91 (350 cycles)	140.8 (initial) 108.7 (350 cycles)
Wu et al. [142]	Cu	Ni_0.8_Co_0.1_Mn_0.1_	2.8–4.3 V RT 1C	145.8 (initial) 127 (100 cycles)	187.9 (initial) 169.4 (100 cycles)
Chen et al. [143]	Ca	Ni_0.8_Co_0.1_Mn_0.1_	3.0–4.3 V RT 0.2C	130 (initial) 86 (50 cycles)	150 (initial) 122 (50 cycles)
Jung et al. [144]	Zr	Ni_0.92_Co_0.04_Mn_0.04_	2.5–4.4 V RT 0.3C	230.1 (initial at 0.1C) 140 (100 cycles)	225.2 (initial) 178 (100 cycles)
Gomez-Martin et al. [145]	Mg	Ni_0.8_Co_0.1_Mn_0.1_	2.8–4.2 V RT 0.33C	195 (initial) 157 (200 cycles)	175 (initial) 140 (600 cycles)
Chu et al. [146]	Ta	Ni_0.6_Co_0.2_Mn_0.2_	3.0–4.5 V RT 1C	180 (initial) 144 (100 cycles)	177 (initial) 152 (100 cycles)
Sattar et al. [147]	Mo	Ni_0.84_Co_0.11_Mn_0.05_	3.0–4.5 V RT 0.5C	209 (initial) 87 (80 cycles)	222 (initial) 172.72 (100 cycles)
He et al. [148]	Na	Ni_0.8_Co_0.1_Mn_0.1_	2.8–4.3 V RT 1C	173.9 (initial) 162.07 (200 cycles)	173.6 (initial) 168.04 (200 cycles)
Yue et al. [149]	F	Ni_0.8_Co_0.1_Mn_0.1_	2.8–4.3 V RT 2C	178 (initial) 140 (100 cycles)	164 (initial) 158 (100 cycles)
Yao et al. [150]	K and Ti	Ni_0.8_Co_0.1_Mn_0.1_	2.75–4.2 V RT 0.2C	195.91 (initial) 166.14 (100 cycles)	193 (initial) 184.72 (100 cycles)
Qiu et al. [151]	W and BO^3−^_3_	Ni_0.92_Co_0.06_Al_0.02_	2.8–4.3 V RT 1C	202.3 (initial) 107.2 (100 cycles)	199.3 (initial) 170.6 (100 cycles)
Chen et al. [152]	BO^3−^_3_ and BO^5−^_4_	Ni_0.8_Co_0.15_Al_0.05_	2.8–4.3 V RT 2C	168.8 (initial) 125.8 (200 cycles)	160.4 (initial) 155.1 (200 cycles)
Zhang et al. [153]	BO^3−^_3_ and BO^5−^_4_	Ni_0.6_Co_0.2_Mn_0.2_	2.8–4.5 V RT 1C	178.2 (initial) 105.4 (100 cycles)	182.2 (initial) 138.6 (100 cycles)

* RT stands for Room Temperature.

**Table 4:** Electrochemical performance of Ni-rich cathode materials before and after coating.

Researchers	Coating	Composition	Testing Conditions	Pristine Material [mAh g^−1^]	Coated Material [mAh g^−1^]
Yang et al. [183]	Mg_3_B_2_O_6_	Ni_0.8_Co_0.1_Mn_0.1_	2.7–4.5 V RT * 1C	204.3 (initial) 108.8 (400 cycles)	200 (initial) 160.6 (400 cycles)
Huang et al. [184]	LiTiO_2_	Ni_0.8_Co_0.1_Mn_0.1_	2.8–4.4 V RT 0.5C	182.6 (initial) 144.26 (100 cycles)	184.5 (initial) 163.47 (100 cycles)
Kong et al. [185]	C-Al_2_O_3_	Ni_0.6_Co_0.2_Mn_0.2_	3.0–4.5 V RT 1C	163 (initial) 133 (100 cycles)	199.58 (initial) 186.6 (100 cycles)
Qian et al. [186]	Li_2_SiO_3_	Ni_0.9_Co_0.05_Mn_0.05_	2.7–4.3 V RT 2C	169.27 (initial) 134.4 (100 cycles)	177.9 (initial) 156.9 (100 cycles)
Du et al. [187]	Li_2_O-2B_2_O_3_	Ni_0.8_Co_0.1_Mn_0.1_	2.75–4.5 V RT 1C	189.1 (initial) 96.0 (100 cycles)	192.0 (initial) 157.7 (100 cycles)
Liu et al. [188]	La_2_Zr_2_O_3_	Ni_0.6_Co_0.2_Mn_0.2_	3.0–4.5 V RT 1C	177.17 (initial) 131.1 (200 cycles)	176.63 (initial) 146.6 (200 cycles)
Wang et al. [189]	AlPO_4_	Ni_0.8_Co_0.1_Mn_0.1_	2.8–4.5 V RT 1C	178.3 (initial) 122.5 (200 cycles)	178.1 (initial) 167.2 (200 cycles)
Zhong et al. [190]	LiFePO_4_	Ni_0.82_Co_0.12_Mn_0.06_	3.0–4.2 V RT 1C	198.25 (initial) 132 (100 cycles)	193.98 (initial) 171 (100 cycles)
Zhu et al. [191]	Li_3_PO_4_	Ni_0.8_Co_0.1_Mn_0.1_	3.0–4.4 V RT 1C	194.89 (initial) 167.8 (100 cycles)	193.85 (initial) 179.5 (100 cycles)
Song et al. [192]	Ca_3_(PO_4_)_2_	Ni_0.8_Co_0.1_Mn_0.1_	3.0–4.3 V 45 °C 1C	210.28 (initial) 83.9 (150 cycles)	213.8 (initial) 155 (150 cycles)
Wang et al. [193]	Li_3_V_2_(PO_4_)_3_	Ni_0.6_Co_0.2_Mn_0.2_	3.0–4.3 V RT 2C	153.7 (initial) 103.9 (200 cycles)	149.6 (initial) 127.1 (200 cycles)
Xiong et al. [194]	LiF	Ni_0.8_Co_0.1_Mn_0.1_	2.8–4.3 V RT 2C	171 (initial) 122.78 (200 cycles)	169 (initial) 138.92 (200 cycles)
Lee et al. [195]	AlF_3_	Ni_0.8_Co_0.15_Al_0.05_	2.7–4.3 V 55 °C 0.5C	197 (initial) 155.827 (200 cycles)	200 (initial) 174.8 (200 cycles)
Dai et al. [196]	CaF_2_	Ni_0.8_Co_0.1_Mn_0.1_	2.7–4.3 V RT 1C	150.4 (initial) 119.0 (200 cycles)	148.2 (initial) 126.5 (200 cycles)
Li et al. [197]	PrF_3_	Ni_0.8_Co_0.1_Mn_0.1_	2.8–4.3 V RT 1C	197.4 (initial) 138.7 (100 cycles)	187.2 (initial) 161.5 (100 cycles)

* RT stands for Room Temperature.

## Data Availability

No new data were created or analyzed in this study. Data sharing is not applicable to this article.
